# Air pollution and depression symptoms in middle-aged and older adults in Los Angeles County

**DOI:** 10.1007/s00420-025-02165-4

**Published:** 2025-08-27

**Authors:** Nicole M. Gatto, Marian Ramzy, Cecilia Rocha, Howard N. Hodis, Fred Lurmann, Victor W. Henderson, Wendy J. Mack

**Affiliations:** 1 Department of Population and Public Health Sciences, Keck School of Medicine, University of Southern California, 1845 N Soto St., Los Angeles, CA 90032 USA; 2 Atherosclerosis Research Unit, Keck School of Medicine, USC, Los Angeles, CA USA; 3 Sonoma Technology, Inc, Petaluma, CA USA; 4 School of Community and Global Health, Claremont Graduate University, Claremont, CA USA; 5 School of Public Health, Loma Linda University, 24951 Circle Dr, Loma Linda, CA 92354 USA; 6 Departments of Epidemiology and Population Health, and of Neurology and Neurological Sciences, Stanford University, Stanford, CA USA

**Keywords:** Air pollution, Depression, Depressive symptoms, CES-D, Particulate matter, PM, Ozone, Nitrogen oxides, Carotid artery intima-media thickness

## Abstract

**Objective:**

Long-term exposure to air pollutants may be harmful to the brain, potentially through inducing oxidative stress or inflammation. Few studies of air pollution and depression have been conducted in the United States where this mental health disorder is prevalent among adults. We investigated associations between ambient air pollutants (O_3_, PM_2.5_ and NO_2_) and depression symptoms in middle-aged and older adults (*n* = 1496) without cardiovascular disease or cognitive impairment in Los Angeles, California.

**Methods:**

Air pollution exposures were assigned to residential addresses using a geographic information system with air quality monitoring data. The Center for Epidemiological Studies-Depression scale (CES-D) assessed depression symptoms at study entry. Carotid artery intima-media thickness (CIMT) was obtained as a measure of subclinical atherosclerosis. Linear and Poisson regression models estimated cross-sectional associations between air pollutants and total CES-D score and suspected clinical depression (CES-D score ≥ 16) adjusting for potential confounders and examined effect modification by CIMT.

**Results:**

Higher exposure to O_3_, PM_2.5_ and NO_2_ overall were not cross-sectionally associated with higher CES-D total scores or CES-D score ≥ 16. However, the interaction between CIMT and PM_2.5_ was statistically significant (β-interaction term = 1.01, 95% CI = 0.05, 1.97; *p*-value = 0.03). Adults with CIMT levels ≥ 0.77 mm had higher depression symptom prevalence as PM_2.5_ increased (β = 0.04 per 10 µg/m^3^, 95% CI = −0.22, 0.30) while those with CIMT < 0.77 mm had lower prevalence (β = -0.18, 95% CI = −0.41, 0.05).

**Conclusions:**

Higher O_3_, PM_2.5_ and NO_2_ exposures were generally unassociated with depressive symptoms. Additional studies are needed to investigate whether persons with higher subclinical atherosclerosis are more susceptible to possible PM_2.5_ effects on mental health.

**Supplementary Information:**

The online version contains supplementary material available at 10.1007/s00420-025-02165-4.

## Introduction

Depression is a common mental health disorder among adults in the United States (US), with national estimates for the years 2013–2016 prior to the COVID-19 pandemic indicating that 8.1% of persons aged 20 years and older experienced moderate levels of depression during a given 2-week period (Brody et al. 2018). In 2014, approximately 1 in 8 people over the age of 12 in the US were currently taking antidepressant medications, representing a 64% increase over 5 years (Pratt and Brody 2017). The underlying pathology of depression is incompletely understood; reductions in monoamines, changes in the hypothalamic-pituitary-adrenal axis (HPA), inflammation, and other mechanisms have been suggested (Malhi and Mann 2018).

In urban regions, ambient air pollution is a mixture of gaseous pollutants and particulate matter (PM) that derive from sources related to motor vehicle fuel combustion, diesel-powered transport and equipment, and local industrial processes (Dickey 2000; Lewtas 2007; Valavanidis et al. 2008). Long-term air pollution exposure (i.e., one month or longer in duration) has consistently been shown to be associated with respiratory (Adam et al. 2015; Schikowski et al. 2014) and cardiovascular diseases (Beelen et al. 2014b; Wang et al. 2014a), and all-cause mortality (Beelen et al. 2014a). Air pollutants may be harmful to the brain, impact cognitive function (Clifford et al. 2016; Gatto et al. 2014; Guxens et al. 2014; Power et al. 2011; Wellenius et al. 2012) and lead to an increased risk of dementia (Chang et al. 2014; Chen et al. 2017; Kioumourtzoglou et al. 2016; Oudin et al. 2016; Power et al. 2016).

Epidemiologic studies of long term air pollution exposure and depression conducted among adult populations in Asia, North America and Europe have provided heterogeneous results (Kioumourtzoglou et al. 2017; Lim et al. 2012; Pun et al. 2017; Shin et al. 2018; Ventriglio et al. 2021; Vert et al. 2017; Wang et al. 2014b, 2018; Zhang et al. 2019; Zijlema et al. 2016). Meta-analyses (Borroni et al. 2022; Braithwaite et al. 2019; Fan et al. 2020; Liu et al. 2021; Zeng et al. 2019; Zhao et al. 2018) which have included some of these studies have reached variable conclusions, but generally show long term exposure to PM_2.5_ to be associated with a 7–12% increase, nitrogen dioxide (NO_2_) a 2–5% increase, and O_3_ no increase in risk of depression, per 10 µg/m^3^ exposure. The findings from four US studies are inconsistent (Kioumourtzoglou et al. 2016; Pun et al. 2017; Qiu et al. 2023; Wang et al. 2014b). One US study suggested possible effect modification between PM_2.5_ exposure and depression and anxiety by cardiovascular comorbidities including stroke and heart failure (Pun et al. 2017). Atherosclerosis is the pathological process underlying cardiovascular disease (Pahwa and Jialal 2023). Populations in US-based studies were exposed to lower levels of air pollution than those in Asia and Europe; thus data are inadequate to compare between exposure levels in the US and other countries. Furthermore, most previous studies in the US (Pun et al. 2017; Qiu et al. 2023; Wang et al. 2014a) (Supplemental Table 1) included only older adults, leaving a gap in information for adults of middle ages. The Los Angeles, California region has some of the highest air pollution levels in the US (American Lung Association 2023). In this study, we investigate cross-sectional associations between residential exposure to O_3_, PM_2.5_ and NO_2_ and depression symptoms among middle-aged and older adults without CVD or cognitive impairment in Los Angeles County.

## Methods

### Study population

We included baseline pre-randomization data from three randomized, double-blinded, placebo-controlled clinical trials (RCTs) conducted at the University of Southern California (USC) Atherosclerosis Research Unit that enrolled healthy, cognitively intact male and female adult participants between 2000 and 2006 [B-Vitamin Atherosclerosis Intervention Trial (BVAIT; ClinicalTrials.gov identifier NCT00114400) (Hodis et al. 2009b), Women’s Isoflavone Soy Health Trial (WISH; NCT00118846) (Henderson et al. 2012), and Early Versus Late Intervention Trial with Estradiol (ELITE; NCT00114517) (Hodis et al. 2015)]. The primary outcome for the three RCTs was reduction in progression of early atherosclerosis, and all RCTs included assessment of depressive symptoms (see below). Briefly, postmenopausal women without clinical evidence of CVD or cognitive impairment were eligible for WISH and ELITE and otherwise healthy men and postmenopausal women with fasting plasma homocysteine levels ≥ 8.5 µmol/L were eligible for BVAIT. Recruitment occurred from Los Angeles County, covering a geographic area of approximately 64,000 km^2^. A total of 8538 individuals were screened across the three trials via telephone or in person; 5367 did not meet eligibility criteria. Reasons for exclusion included clinical signs or symptoms of CVD, diabetes mellitus or fasting serum glucose ≥ 126 mg/dL, triglyceride (TG) levels ≥ 500 mg/dL, hypertension [systolic blood pressure (SBP) ≥ 160 mmHg and/or diastolic blood pressure (DBP) ≥ 100 mmHg)], untreated thyroid disease, creatinine clearance < 70 ml/min or serum creatinine > 2.0 mg/dL, a life threatening disease with prognosis < 5 years, alcohol intake > 5 drinks per day/substance abuse, unwillingness to stop taking B-vitamin supplements (BVAIT), current use of hormone therapy (WISH or ELITE), hysterectomy and no oophorectomy (ELITE), or 6–9 years postmenopausal (ELITE). Of the 1509 subjects who were randomized, 13 did not have data on air pollution exposure (*n* = 9) or depression symptoms (*n* = 4) leaving 1496 (99.1%) participants included in this study.

All human research was approved by the USC Institutional Review Board (WISH Approval#: HS-035001; BVAIT Approval#: HS-98B012; ELITE Approval#: HS-04A024), and all participants provided written informed consent.

### Measurements

#### Assessment of air pollution exposure

Air pollution exposure assignments were derived from measured ambient air quality data spatially mapped to participants’ geocoded residence addresses using a geographic information system (GIS). These data were initially automatically geocoded to TigerLine files (Navteq, 2006), then manually resolved in a multi-step process similar to that described by McElroy (McElroy et al. 2003). Ambient air quality data were primarily extracted from the Air Quality System (AQS), maintained by the US Environmental Protection Agency (http://www.epa.gov/ttn/airs/airsaqs/). A database of O_3_ [8-h maximum, in parts per billion (ppb)], NO_2_ (24 h, in ppb) and PM_2.5_ (24 h, in µg/m^3^) concentrations at monitoring stations was compiled from a June 2008 AQS version. Measurements obtained using Federal Reference Methods and Federal Equivalent Methods were included and supplemented with monthly average O_3_, NO_2_, and PM_2.5_ concentrations measured in the Southern California Children’s Health Study (Peters et al. 2004). Daily, monthly, and annual average concentrations were calculated using a 75% data completeness criterion. The database included measurements from California and border areas of nearby states for calendar years 2000–2006. The density of measurement stations in this regional air monitoring network was every 20–40 km in urban areas and 50 to 150 km in rural areas (ARB 2008).

Annual daily average concentrations of air pollutants from monitoring stations during 2000–2006 were spatially interpolated to participants’ residential addresses using inverse-distance-squared weighting. Specifically, if one or more stations with valid data for a specific year were located within 5 km of a residence, the air pollutant assignment was based solely on local data. If there were no stations within 5 km, air pollutant assignments were calculated from the three closest stations with valid data located within 100 km of the residence. Historical address data were not available for participants for years prior to their enrollment in the RCTs. To derive measures of long-term residential exposure to O_3_, NO_2_, and PM_2.5_, we averaged estimated air pollution exposure at the residential address where the participant lived at enrollment in the RCT and at that address for the prior year. Air pollution exposure was scaled to 10 parts per billion (ppb) for O_3_ and NO_2_, and to 10 ug/m^3^ for PM_2.5_.

#### Depressive symptoms

The Center for Epidemiological Studies-Depression scale (CES-D) (Radloff 1977) is a 20-item measure of current depressive symptomatology. The CES-D queries the frequency of symptoms such as restless sleep, poor appetite, and feeling lonely over the previous week. Response options range from 0 to 3 for each item (0 = Rarely or None of the Time, 1 = Some or Little of the Time, 2 = Moderately or Much of the time, 3 = Most or Almost All the Time). Total CES-D scores range from 0 to 60, with higher scores indicating greater risk for depression. We used the CES-D assessment obtained at the baseline visit for each participant. Participants were also categorized in two groups using a score of ≥ 16 following commonly recommended practices to identify individuals with suspected prevalent clinical depression (Lewinsohn et al. 1997; Vilagut et al. 2016; Weissman et al. 1977).

#### Antidepressant medications

Information on current medication use was self-reported by participants on questionnaires prior to trial randomization and administration of the CES-D. All records were reviewed by one research assistant (MR) for reports of drugs used to treat the symptoms of clinical depression as defined by Anatomical Therapeutic Chemical (WHO 1993) classification code N06A including non-selective monoamine reuptake inhibitors, selective serotonin reuptake inhibitors, non-selective monoamine oxidase inhibitors, monoamine oxidase A inhibitors, and other antidepressants. We considered participants to have antidepressant medication use if they reported one or more of the medications from the listed classes.

#### Sociodemographic and covariate data

All participants completed a questionnaire regarding sociodemographic factors, smoking status, and a 7-day physical activity recall that elicited number of hours spent in different activities including sleep. Each reported physical activity was converted to metabolic equivalents (METs) and MET-hours (METs times duration of activity); a summary variable accumulated MET-hours over all reported activities. Physical exam measures included blood pressure, body height and weight; body mass index (BMI) was calculated (weight in kg/height in m^2^). Blood samples were drawn after a minimum 8-h fasting period. Total cholesterol, high-density lipoprotein cholesterol (HDL- cholesterol) and low-density lipoprotein cholesterol (LDL- cholesterol, calculated) were measured by standardized enzymatic assay methodology (Lipid Clinics Research Program, 1974). Fasting serum glucose levels were measured using the glucose oxidase technique on a Beckman Glucose II analyzer (Beckman Instruments, Brea, CA, USA).

Carotid artery intima-media thickness (CIMT) is a measure of subclinical atherosclerosis and an independent predictor of cardiovascular risk (Bauer et al. 2012). The right common carotid artery was imaged using high resolution B-mode ultrasound with simultaneously recorded single-lead electrocardiogram for cardiac gating (Hodis et al. 2001, 2009a, 2011). For the primary trial outcome, CIMT was measured at the distal common carotid artery far wall during minimum lumen diameter (approximate diastole) with in-house developed automated computerized edge detection software (patents 2005, 2006, 2011) (Hodis et al. 2001; Selzer et al. 2001). CIMT was determined as the average of approximately 70–100 individual measurements between the intima–lumen and media–adventitia interfaces along a 1-cm length just proximal to the carotid artery bulb. This method standardizes the location, distance, and timing over which CIMT is measured and ensures that the same portion of arterial wall is measured in each image and compared within and across all participants. The coefficient of variation for repeated baseline CIMT measurements is < 1% (Hodis et al. 2009b). Using the mean for the study population, we classified participants as having average and higher (≥ 0.77 mm) or lower than average (< 0.77 mm) CIMT levels.

### Statistical analysis

We examined two outcomes in regression models (1) total CES-D score as a measure of prevalent depressive symptoms, and (2) suspected prevalent clinical depression indicated by a CES-D score ≥ 16. Linear regression methods with continuous scaled terms for individual air pollution measures were used to estimate cross-sectional associations with total CES-D score. Because CES-D scores were positively skewed in the study population, we applied a log transformation of the total CES-D score in the linear models and back transformed results for interpretation. We used robust Poisson regression models (Barros and Hirakata 2003; Zou 2004) to estimate cross-sectional associations with air pollution exposures and suspected prevalent clinical depression (yes, no).

Model covariables were selected a priori based on findings from previous studies (Kioumourtzoglou et al. 2017; Pun et al. 2017; Qiu et al. 2023; Wang et al. 2014b). Simple models were adjusted for sociodemographic characteristics including age (continuous), race/ethnicity (Asian, Pacific Islander, Native American; Black; Hispanic/Latino; non-Hispanic White), sex, educational level (high school or less, some college, Bachelor’s degree, graduate/professional degree), household income (< 30,000, 30,000–49,999, 50,000–69,999, 70,000–99,999 and ≥ 100,000 US dollars/year), and indicator variables for year and study (BVAIT, WISH, ELITE). Multivariable models were adjusted for covariables from simple models plus BMI (< 25, 25–29.9, ≥ 30 kg/m^2^), smoking status [ever (former/current), never], physical activity (total MET-hours), fasting glucose, systolic blood pressure, LDL-cholesterol, HDL-cholesterol and anti-depressant medication use (yes, no).

We tested interaction terms individually for O_3_, NO_2_, and PM_2.5_ by sex and CIMT level (continuous) in multivariable models using a threshold of *p*-value < 0.05 for effect modification. We used the categorized CIMT variable (< 0.77 mm, ≥ 0.77 mm) in stratified models to estimate air pollution-depression associations by CIMT level. Because air pollutants share common sources, we conducted two-pollutant multivariable models for O_3_ and NO_2_, or O_3_ and PM_2.5_ in order to explore the joint effects and contributions of individual pollutants in combination with one other pollutant on symptoms of depression. Because NO_2_ and PM_2.5_ exposures were highly correlated in our study population (Supplemental Table 2), we did not estimate a joint effects model for these two pollutants. In sensitivity analyses, we expanded the grouping of participants with suspected prevalent clinical depression to include those using medications prescribed for depression or having a total CES-D score ≥ 16. In additional sensitivity analyses, we included an indicator variable for annual unemployment rate for Los Angeles County to examine the effect of controlling for a macro-level economic factor in the multivariable models of CES-D total score. The annual unemployment rate for Los Angeles County between 2000 and 2006 was categorized as low (≤ 5.3%), average (5.4–5.8%) or high (≥ 5.9%) unemployment. We report beta coefficients (β) with 95% confidence intervals (CI) from linear regression models, and risk ratios (RRs) with 95% CIs from Poisson regression models per 10 units of the air pollutants.

All analyses used SAS version 9.4 (SAS Institute Inc., Cary, NC, USA.).

## Results

**Fig. 1:**
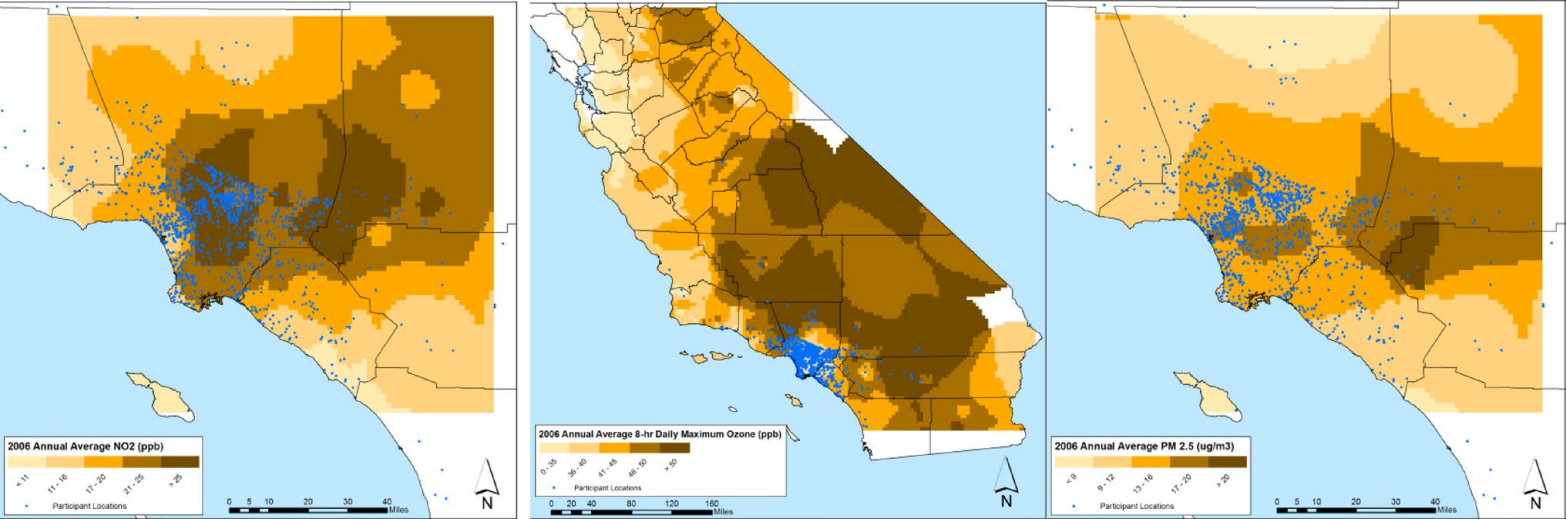
*Geographic variability in exposure to air pollution (O_3_, NO_2_, PM_2.5_) in Southern California in 2006 with Study Participants’ Addresses. *reproduced from Gatto et al. 2014

**Fig. 2:**
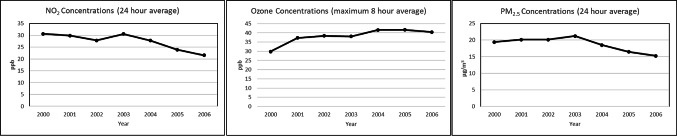
Temporal variability in exposure to air pollution (O_3_, NO_2_, PM_2.5_) Estimated at Study Participants’ Residences in Southern California Between the Years 2000–2006

The mean (± SD) age of the study population was 60.5 (± 8.1) years; 1,84 (79.4%) were women and 914 (61.1%) had a Bachelor’s degree or higher (Table 1). Annual average levels of O_3_, NO_2_ and PM_2.5_ air pollution varied across the Southern California region where study participants’ residential addresses were located (Fig. 1 shows the year 2006) as well as temporally across the years 2000–2006 (Fig. 2). The median [inter-quartile range] total CES-D score was 5 [2, 11], 12.9% of participants had suspected prevalent clinical depression (defined as a CES-D score ≥ 16), and 210 (14%) self-reported a drug prescribed for depression. Proportionally more women than men had a CES-D score ≥ 16 (14.9% vs. 5.2%, respectively, *p* < 0.0001) or reported a depression medication (16.1% vs. 6.2%, respectively, *p* < 0.0001).

**Table 1:** Characteristics of study population (*n* = 1496)

Variable, mean ± SD or number (%)	Overall *n* = 1496 (100%)	CESD < 16 *n* = 1303 (87.1%)	CESD ≥ 16 *n* = 193 (12.9%)
Age (years)	60.5 ± 8.1 (range: 40–92)	60.7 ± 8.1 (40–92)	58.9 ± 7.7 (41–83)
40–54	347 (23.3)	291 (22.4)	56 (29.2)
55–59	358 (24.0)	305 (23.5)	53 (27.6)
60–64	351 (23.5)	310 (23.9)	41 (21.4)
≥ 65	436 (29.2)	394 (30.3)	42 (21.9)
Sex			
Female	1,184 (79.4)	1,008 (77.5)	176 (91.7)
Male	308 (20.6)	292 (22.5)	16 (8.3)
Race/Ethnicity			
Asian, Pacific Islander, Native American	146 (9.8)	128 (9.9)	29 (15.1)
Black	156 (10.5)	136 (10.5)	20 (10.4)
Hispanic or Latino	201 (13.5)	172 (13.2)	29 (15.1)
Non-Hispanic White	989 (66.3)	864 (66.5)	125 (65.1)
Educational level			
High school or less	140 (9.4)	117 (9.0)	23 (11.9)
Some college	407 (27.2)	344 (26.4)	63 (32.6)
Bachelor’s degree	375 (25.1)	325 (24.9)	50 (25.9)
Graduate/professional degree	539 (36.0)	485 (37.2)	54 (28)
Other	35 (2.3)	32 (2.5)	3 (1.6)
Household income (US dollars/year)			
< 30,000	283 (18.9)	228 (17.5)	55 (28.5)
30,000–49,999	223 (14.9)	189 (14.5)	34 (17.6)
50,000–69,999	310 (20.7)	273 (21.0)	37 (19.2)
70,000–99,999	261 (17.5)	240 (18.4)	21 (10.9)
≥ 100,000	419 (28.0)	373 (28.6)	46 (23.8)
Marital status			
Single	128 (8.6)	102 (7.9)	26 (13.6)
Married/Domestic partner	886 (59.5)	791 (60.9)	95 (49.7)
Separated, divorced, widowed	476 (31.9)	406 (31.3)	70 (36.7)
Study			
BVAIT	506 (33.8)	463 (35.5)	43 (22.3)
WISH	348 (23.3)	308 (23.6)	40 (20.7)
ELITE	642 (42.9)	532 (40.8)	110 (57.0)
Body-mass index (kg/m^2^)	27.3 ± 5.2	27.2 ± 5.1	28.4 ± 5.9
≤ 25	518 (34.9)	458 (35.3)	60 (31.8)
25–30	582 (39.2)	522 (40.3)	60 (31.8)
> 30	386 (26.0)	317 (24.4)	69 (36.5)
Smoking status			
Never smoker	903 (60.6)	804 (61.9)	99 (51.6)
Current or former smoker	588 (39.4)	495 (38.1)	93 (48.4)
Blood Pressure (mmHg)			
Systolic	121.3 ± 16.0	121.5 ± 16.1	119.9 ± 15
Diastolic	76.7 ± 9.7	76.8 ± 9.8	75.8 ± 9.1
CIMT (mm)	0.77 ± 0.12	0.78 ± 0.13	0.75 ± 0.1
Cholesterol			
Low density lipoprotein-cholesterol	137.6 ± 34.1	137.4 ± 34.0	139.1 ± 34.7
High density lipoprotein-cholesterol	62.2 ± 17.3	62.2 ± 17.5	62.2 ± 16.1
Glucose (mg/dl)	96.7 ± 10.8	96.8 ± 10.7	96.2 ± 11.4
Total METs per week	247.3 ± 22.7	247.8 ± 22.9	243.6 ± 20.8
Hours of sleep per week	48.5 ± 7.9	48.8 ± 7.5	46.0 ± 10.3
CES-D total score, median [IQR]	5 [2, 11]	4 [1, 8]	20 [17, 25]
Use of medications prescribed for depression			
No	1286 (86.0)	1143 (87.7)	143 (74.1)
Yes	210 (14.0)	160 (12.3)	50 (25.9)

SD: Standard deviation

IQR: Inter-quartile range

CIMT: carotid artery intima-media thickness

METs: metabolic equivalents

CES-D: Center for Epidemiological Studies-Depression scale

BVAIT: B-Vitamin Atherosclerosis Intervention Trial

WISH: Women’s Isoflavone Soy Health Trial

ELITE: Early Versus Late Intervention Trial with Estradiol

After adjusting for sociodemographic characteristics, smoking status, BMI, physical activity level, fasting glucose, systolic blood pressure, LDL-cholesterol, HDL-cholesterol and anti-depressant use, increasing exposure to PM_2.5_ was not associated overall with higher total CES-D scores (β = −0.10 per 10 µg/m^3^ 95% CI = −0.27, 0.07) (Table 2). However, the interaction between CIMT and PM_2.5_ was statistically significant (β-interaction term = 1.01, 95% CI = 0.05, 1.97; *p*-value = 0.04). Models stratified by CIMT level indicated that the association between PM_2.5_ and total CES-D score was negative among persons with CIMT < 0.77 mm (β = −0.18, 95% CI = −0.41, 0.05) and positive among persons CIMT ≥ 0.77 mm (β = 0.04, 95% CI = −0.22, 0.30), yet confidence intervals for neither result excluded the null. Higher exposure to NO_2_ or O_3_ were not associated with higher CES-D total scores (Table 2) and there was no effect modification by sex or CIMT (*p*-values for the interaction > 0.05). Increasing exposure to O_3_, NO_2_ or PM_2.5_ did not associate with a higher relative risk of suspected clinical depression defined as a CES-D ≥ 16, or when the definition was expanded to include reported use of anti-depressants in sensitivity analyses. There was no effect modification by sex or CIMT for exposure to any of the three pollutants and suspected clinical depression (*p*-value for interaction > 0.05). O_3_, NO_2_ and PM_2.5_ air pollution-depression estimates were slightly attenuated in models that additionally accounted for a second pollutant, but conclusions were unchanged. Including an indicator variable for annual unemployment rate for Los Angeles County in our multivariable model of CES-D total score resulted in minor attenuations of βs for O_3_, NO_2_, and PM_2.5_ towards the null and conclusions were unchanged.

**Table 2:** Associations between levels of ambient air pollutants and depression symptoms or suspected clinical depression from regression models

Air Pollutant	Simple^a^	Multivariable^b^	Two-pollutant^c^	
CES-D total score (β, 95% CI)^d^				
O_3_, per 10 ppb	0.07 (−0.02, 0.16)	0.05 (−0.04, 0.15)	0.04 (−0.06, 0.14)	0.04 (−0.06, 0.14)
NO_2_, er 10 ppb	−0.05 (−0.13, 0.04)	−0.04 (−0.12, 0.04)	–	−0.02 (−0.11, 0.07)
PM_2.5_, per 10 µg/m^3^	−0.12 (−0.29, 0.05)	−0.10 (−0.27, 0.07)	−0.07 (−0.26, 0.12)	–
Suspected clinical depression (CES-D ≥ 16) (RR, 95% CI)^e^				
O_3_, per 10 ppb	1.03 (0.78, 1.37)	0.99 (0.74, 1.32)	0.95 (0.70, 1.29)	0.95 (0.70, 1.30)
NO_2_, per 10 ppb	0.91 (0.71, 1.18)	0.92 (0.71, 1.18)		0.90 (0.68, 1.19)
PM_2.5_, per 10 µg/m^3^	0.83 (0.49, 1.40)	0.85 (0.50, 1.44)	0.82 (0.47, 1.44)	
Suspected clinical depression (CES-D ≥ 16) or use of anti-depressant medications (RR, 95% CI)^e^				
O_3_, per 10 ppb	1.14 (0.93, 1.40)	0.96 (0.78, 1.19)	0.92 (0.73, 1.16)	0.91 (0.72, 1.15)
NO_2_, per 10 ppb	0.89 (0.74, 1.07)	0.93 (0.77, 1.11)	–	0.90 (0.73, 1.09)
PM_2.5_, per 10 µg/m^3^	0.72 (0.50, 1.04)	0.87 (0.59, 1.27)	0.82 (0.54, 1.23)	–

CES-D: Center for Epidemiological Studies-Depression scale

β: beta coefficient

RR: rate ratio

CI: confidence interval

^a^ Simple models are adjusted for age (continuous), race/ethnicity (non-Hispanic White; Black; Hispanic/Latino; Asian, Pacific Islander, Native American), sex, educational level (high school or less, some college, Bachelor’s degree, graduate/professional degree), household income (< 30,000, 30,000–49,999, 50,000–69,999, 70,000–99,999 and ≥ 100,000 US dollars/year), year, study (BVAIT, WISH, ELITE)

^b^ Multivariable models are adjusted for variables in the simple model plus BMI (< 25, 25-29.9, ≥ 30), smoking status (ever, never), physical activity (total MET hours), glucose, SBP, LDL-cholesterol, HDL- cholesterol, use of medications prescribed for depression (yes, no)

^c^ Two pollutant models (O_3_ and NO_2_; O_3_ and PM_2.5_); adjusted for variables in multivariable models

^d^ From linear regression models, *n* = 1,459

^e^ From Poisson regression models, *n* = 1,481

## Discussion

Population growth in the US continues to shift to urban metropolitan areas (Perry et al. 2000), and climate change is expected to increasingly impact levels and locations of ambient air pollutants such as ground-level O_3_ and PM (Clayton 2021; USGCRP et al. 2018). Therefore, it is important to understand possible mental health effects from air pollution exposure. In this study of 1496 middle-aged and older healthy adults without cardiovascular disease or cognitive impairment residing in the Los Angeles, California area, our results did not suggest that higher levels of residential exposure to PM_2.5_, O_3_ or NO_2_ air pollution between 2000 and 2006 were associated with higher CES-D scores overall or suspected prevalent clinical depression. Our conclusions were not changed when we took into consideration the joint effects of air pollutants reflecting exposure mixtures in Southern California or when we considered an expanded definition of suspected clinical depression to include reported use of anti-depressant medications. For PM_2.5_, we observed that the direction of the association with total CES-D scores varied by CIMT level, which may suggest effect modification by subclinical atherosclerosis.

Four published studies of long-term air pollution exposure and depression have been conducted among adult populations in the US (Kioumourtzoglou et al. 2017; Pun et al. 2017; Qiu et al. 2023; Wang et al. 2014b). Wang et al. concluded that there was no positive association between the presence of depressive symptoms and long term exposure (i.e., averaged over 365 days) to traffic pollution estimated by distance to nearest roadway or outdoor black carbon levels (Wang et al. 2014b). Pun et al. reported odds ratios > 1 for current moderate-to-severe depression symptoms with one-year (OR = 1.06; 95% CI 0.89, 1.27) and four-year exposure (OR = 1.14; 95% CI 0.97, 1.34) to PM_2.5_, yet confidence intervals for both estimates included the null (Pun et al. 2017). In our study population, average exposure to PM_2.5_ was higher compared with either the MOBILIZE or NSHAP populations, and average exposure to NO_2_ and O_3_ was higher than the MOBILIZE population. Despite these higher relative exposures, air quality in California has improved during the last three decades because of air pollution regulation and emission control strategies (California Air Resources Board ; Lurmann et al. 2015). In the Southern California region for the years 2000–2006 covered by our study, trends in annual air pollution levels were generally downward for NO_2_ and PM_2.5_ and flat for O_3_ (Lurmann et al. 2015).

Kioumourtzoglou et al. (Kioumourtzoglou et al. 2017) reported non-statistically significantly elevated hazard ratios for antidepressant use or physician diagnosis of depression among women for summer O_3_ exposure (HR = 1.06 per 10 ppb; 95% CI 1.0, 1.12) and for 1-, 2- or 5-year PM_2.5_ exposure [HRs = 1.08 (0.97, 1.2), 1.08 (0.97, 1.2), 1.07 (0.95, 1.19) per 10 ug/m^3^, respectively]. Associations were not changed when adjusted for cardiovascular disease-related variables. Qiu et al. (Qiu et al. 2023) found statistically significant elevations in later life depression diagnoses from five-year exposure to PM_2.5_ (HR = 1.021; 95% CI 1.013, 1.030) and O_3_ (HR = 1.023; 95% CI 1.018,1.028) per 5-unit increases of the pollutants among Medicare enrollees. Reported average O_3_ exposure among Medicare enrollees was more comparable to levels in our study population than were those among the Nurses’ Health Study and MOBILIZE study. The Qiu et al. research is the only other available US study to provide data on associations between depression and long-term exposure to NO_2_ (HR = 1.008; 95% CI 1.005, 1.011 per 5-unit increase).

In contrast to previous US-based studies, the mean age of our study population was 6.6–18.1 years younger. Thus, we contribute data on air pollution-depression associations at a lower range of the age spectrum in older adults, as well as at the highest air pollution exposure levels among US studies. Exclusion criteria of the parent clinical trials from which our data were derived established study populations that were free of clinical cardiovascular disease, cognitive impairment, diabetes and hypertension. Thus, our results reflect air pollution effects among a relatively healthier population than those included in the MOBILIZE, NSHAP, Nurses’ Health and Medicare Enrollees studies. The research among the Nurses’ Health Study and Medicare enrollees included much larger numbers of participants than our own research and thus had greater power to detect small effects.

Studies conducted where air pollution levels are generally higher than in the US indicate that PM_10_ (Lim et al. 2012; Shin et al. 2018; Zhang et al. 2019), O_3_ (Lim et al. 2012) and NO_2_ (Lim et al. 2012; Shin et al. 2018; Vert et al. 2017; Zijlema et al. 2016) are associated with incident and prevalent depression measured by questionnaire, self-report of physician diagnosis, or medication use, while results for PM_2.5_ have been contradictory (Wang et al. 2018; Zhang et al. 2019).

Measurement error is a concern for air pollution studies because pollution levels vary spatially and temporally. While exposure assignments in our research were derived from a comprehensive air monitoring system in the US where monitoring stations are typically located 20 to 30 km apart, pollutant concentrations vary on smaller spatial scales than can be resolved with regional air monitoring networks. Nevertheless, a strength of our exposure assessment was the availability of data for multiple pollutants with fine spatial resolution across the study region. Furthermore, our analyses accounted for sociodemographic, lifestyle and metabolic factors and use of medications prescribed for depression. The cross-sectional nature of the analysis is a limitation as historical residential address data for participants were not collected and we could not estimate air pollution exposure retrospectively beyond the prior year. Because no children or only postmenopausal women were included as participants, the generalizability of our results is limited. A strength of our study was the standardized, validated instrument administered to all participants to assess depressive symptoms. In addition to personal economic circumstances, local, state and national (“macro”) economic factors could relate to both regional air quality (Wang and Liu 2025) and individuals’ psychological outlook (Puri and Robinson 2007). During periods of greater economic activity, urban air pollution may increase due to more production at factories (stationary sources) and greater movement of employed persons to and from work and transport of goods (mobile sources) (Davis 2012). People’s moods may also reflect optimism from strong employment and stable household income (Brenner and Bhugra 2020; Meltzer et al. 2010; Zivin et al. 2011). Including an indicator variable for annual unemployment rate for Los Angeles County did not change our conclusions of the associations between air pollution and CES-D total score. Additional studies to disentangle the role of economic factors in research of ambient air pollution and population mental health are needed.

Processes through which CVD may have effects on brain health could also link PM pollution to mental health outcomes (Breton et al. 2012, 2016; Kunzli et al. 2005, 2010). Cardio-/cerebro-vascular disease can result in reduced blood flow and diminished delivery of oxygen to the brain (Crowley 2004). Possible effect modification by CIMT of the relationship between PM and depression symptoms should be further investigated. It is plausible that adults with higher CIMT may be more susceptible to the effects of PM. Previous epidemiologic research has shown stroke, heart failure and hypertension to be effect modifiers of PM-mental health associations (Pun et al. 2017). However, it is unclear what could explain the inverse relationship between PM_2.5_ and CES-D total score among adults with lower CIMT levels in our study. The inverse association may have been due to another factor that was not accounted for in analyses, or a chance finding since the results failed to exclude the null.

In conclusion, higher O_3_, PM_2.5_ and NO_2_ exposures were generally unassociated with depressive symptoms. Additional research should investigate whether persons with higher subclinical atherosclerosis are more susceptible to possible PM_2.5_ effects on mental health. Large studies of long-term ambient air pollution exposure that examine the multiple components of air pollution and include other measures of depression are needed among US populations. These will further inform public health recommendations and environmental air quality policy or legislation as urbanization in the US continues.

## Supplementary Information

Below is the link to the electronic supplementary material.

Supplementary Material 1

## Data Availability

Data for the current study are not publicly available to protect study participant privacy.
