# Supplementary material for: Air pollution and depression symptoms in middle-aged and older adults in Los Angeles County
Source: Int Arch Occup Environ Health. 2025 Aug 27;98(8):731–41. doi: 10.1007/s00420-025-02165-4 (PMC12494606; doi:10.1007/s00420-025-02165-4)
Supplement: Supplementary file 1 — Supplementary Material 1 [file 420_2025_2165_MOESM1_ESM.docx]

**Supplemental Table 1. Epidemiologic studies of long-term residential exposure to air pollution in the United States and depression, by date of publication and size of study population**

| **First author, Publication date** | **Design, statistical analysis approach** | **Study Population** | **Exposure/How estimated** | **Average Air Pollution Levels** | **Outcome/How Measured** | **Covariates** | **Findings** |
| --- | --- | --- | --- | --- | --- | --- | --- |
| Wang, 2014 | Prospective Cohort; repeated measures | 732 adults from MOBILIZE Boston study  ages ≥ 65 years  2005-2008 | Traffic pollution/distance to nearest roadway  Annual black carbon (BC) levels  Spatio-temporal land-use regression model | NO_2_: 16.7 ppb  O_3_: 24.5 ppb  PM_2.5_: 8.6 μg/m^3^ | Depressive symptoms/  CESD-R | • Basic model: age, sex, race/ethnicity, visit, season, household income, education, neighborhood SES  • Second model: plus BMI, physical activity, alcohol intake, smoking, diabetes, hypertension, hyperlipidermia, antidepressant use | No association between distance to nearest roadway or mean annual BC concentration and presence of depressive symptoms |
| Pun, 2017 | Longitudinal Study, GLM | 4,008 adults across the  US from National Social Life, Health, and Aging Project (NSHAP)  ages 57-85 years  2005-2011 | PM_2.5_ at 180 days, 365 days, 4 years  Spatiotemporal generalized  additive mixed models with US EPA data | PM_2.5_: 11.1 μg/m^3^ | Depressive symptoms (moderate to severe)/  CESD-11 | • Basic model: age, sex, race/ ethnicity, year, season, day of week, residence within metropolitan statistical area  • Multivariable model: plus educational attainment, family income, median household income, % below poverty level in census tract  • Wave-specific: plus BMI, smoking, physical activity, alcohol intake, loneliness, current use of antidepressant, history of diabetes, hypertension, stroke, heart failure, emphysema, COPD, or asthma | Per 5 μg/m^3^ PM_2.5_  180 days: OR=1.04 (95% CI: 0.89, 1.22)  365 days: OR=1.06 (95% CI: 0.89, 1.27)  4 years: OR=1.14 (95% CI: 0.97, 1.34)  Effects were stronger when restricted to Wave 2 participants who did not have depression in Wave 1 |
| Kioumourtzoglou, 2017 | Prospective Cohort; Cox regression | 41,844 women in Nurses' Health Study  ages 30-55 years  1996-2008 | 1, 2, and 5-year average PM_2.5_; Average O_3_ in summer months  National  spatiotemporal model (PM_2.5_) & US EPA data (O_3_) weighted square distances | O_3_ (summer): 31.9 ppb  PM_2.5_ (1 year): 12.6 μg/m^3^ | Depression onset/physician diagnosis or use of anti-depressant medication | Calendar year and month, census region, living in Metropolitan Statistical Area, individual-level SES, community-level SES, race, physical activity, BMI, pack-years of smoking, smoking status, dietary habits, multivitamin intake, participation in social groups, baseline abbreviated Mental Health Inventory Score | Per 10-ppb O_3_: HR= 1.06 (95% CI: 1.00, 1.12) for antidepressant use or depression diagnosis  HR=1.08 (95% CI: 1.02, 1.14) for use of antidepressant medication  NS depression diagnosis  Per 10-μg/m^3^ PM_2.5_ at 1- year: HR=1.12 (95% CI: 1.00, 1.25) for use of antidepressant medications  NS 2- and 5-year  NS 1-, 2-, and 5-year for antidepressant use or depression diagnosis, depression diagnosis  No change when adjusting for CV-related variables |
| Qiu, 2023 | population-based longitudinal cohort study; Cox regression | 8,907,422 Medicare enrollees  ages ≥ 65 years  2005-2016 | Current and past 5-year PM_2.5_, NO_2_, O_3_  high-performance national air pollution models (zip code level) | PM_2.5_, μg/m^3^ mean (SD) 9.6 (2.7)  NO_2_, ppb, median (IQR) 15.4 (10.4-23.1)  O_3_, ppb, mean (SD) 39.1 (4.0) | late life depression diagnosis/identified from Medicare claims | Calendar year, region, temperature, precipitation, Vegetation Index, population density, community poverty, low education rate, nearest hospital distance, % ambulatory visit level, % Blacks, % Asians, % Hispanics, community smoking rate, % renting, and income extreme index (community psychosocial stress proxy) | Per 5-unit increase in long term mean pollutant: single [tri] pollutant models  PM_2.5_ HR=1.021, 95% CI: 1.013-1.030 [1.009 (1.000-1.018)]  NO_2_ HR= 1.008, 95% CI: 1.005-1.011 [1.006 (1.003-1.009)]  O_3_ HR= 1.023, 95% CI: 1.018-1.028  [1.021 (1.016-1.026)]  Older adults with comorbidities more sensitive to NO_2_ |

**Supplemental Table 2. Average annual daily levels of ambient air pollutants estimated at residential addresses in Los Angeles County of study participants** **during 2000-2006, and correlations between pollutants**

| **Pollutant** | **Mean** | **Median** | **SD** | **Minimum** | **Maximum** | **IQR** | **r** | | |
| --- | --- | --- | --- | --- | --- | --- | --- | --- | --- |
|  |  |  |  |  |  |  | **NO_2_** | **O_3_** | **PM_2.5_** |
| NO_2_, ppb (24-hour) | 25.2 | 25.7 | 6.7 | 3.9 | 40.9 | 9.4 | - | -0.47 | 0.81 |
| O_3_, ppb (8-hour maximum) | 39.9 | 39.9 | 5.4 | 21.3 | 58.9 | 5.9 |  | - | -0.45 |
| PM_2.5_, μg/m^3^ (24-hour) | 17.3 | 16.8 | 3.6 | 5.6 | 28.1 | 4.9 |  |  | - |

ppb: parts per billion

SD: Standard deviation

IQR: Inter-quartile range
